# Oral and oropharyngeal squamous cell carcinoma in young adults: A retrospective study in Granada University Hospital

**DOI:** 10.4317/medoral.21755

**Published:** 2017-10-21

**Authors:** Paolo Cariati, Almudena Cabello-Serrano, Miguel Perez-de Perceval-Tara, Fernando Monsalve-Iglesias, Ildefonso Martínez-Lara

**Affiliations:** 1 Oral and Maxillofacial surgery resident. Hospital Universitario Virgen de las nieves, Granada, Spain; 2 Head of the Oral and Maxillofacial Surgery Service of the Hospital de Granada. Hospital Universitario Virgen de las nieves, Granada, Spain

## Abstract

**Background:**

This study aims to evaluate and analyze the clinical features and outcomes of oral and oropharyngeal squamous cell carcinoma (SCC) in patients < 45-years old in our center.

**Material and Methods:**

A retrospective analysis was conducted using the records of patients diagnosed with oral and oropharyngeal SCC between 1998 and 2011 in the University Hospital of Granada (Spain). The analysis identified 33 patients with oral and oropharyngeal SCC with an age of <45 years. Moreover, during the years studied, a further 472 patients were diagnosed with oral and oropharyngeal SCC in our center. Thus, 100 SCC patients with an age of >45 years were randomly selected from the same database. A retrospective analysis was conducted to determine specific features including sites of occurrence, risk factors, sex distribution, socio-economic status, T stage at diagnosis, nodal involvement, degree of tumor differentiation, locoregional failure and overall survival at 5 years was. Further, the results of both groups were compared.

**Results:**

The male-female ratio was 1.2:1 in the group of young adults and 2.03:1 in the group of patients with an age of >45 years. No significant differences were found in terms of site, nodal involvement, locoregional failure, and overall survival. However, there were statistically significant differences between the two groups in terms of features such as risk factors, socio-economic status, T stage at diagnosis, and degree of tumor differentiation. The overall 5-year survival rate was 62% for patients >45 years old, whilst for the group of young adults this rate was 48.4% (*p*= 0.17).

**Conclusions:**

The poor association between the common risk factors and oral and oropharyngeal cancers in young adults suggests that other pathogenic mechanisms should be investigated. For young patients, the data show evidence of poorer outcomes in terms of overall survival (*p*=0.17), and locoregional failure (*p*=0.23). Nevertheless, the literature shows that the results in this field are particularly inconsistent, and further research is therefore needed to provide more in-depth knowledge of the disease in this age group.

** Key words:**Oral and oropharyngeal squamous cell carcinoma, young adults, poor prognosis,risk factors.

## Introduction

Head and neck squamous cell carcinoma (HNSCC) is the sixth most common cancer worldwide. It represents 5-6% of all cancers and usually occurs during the sixth and seventh decades of life (1,2). The use of tobacco and alcohol are strongly associated with the occurrence of this malignancy (3). Unfortunately, despite progress in treatment protocols the prognosis still remains poor (4). Furthermore, several reports have described an increased incidence of HNSCC in young people (5). This is particularly true for certain locations such as the oral cavity (6).

The key reason for this epidemiological change is unknown. Interestingly, numerous authors have confirmed that the association between oral and oropharyngeal SCC and use of alcohol and tobacco is less evident in young people (7). In particular, the results of various studies suggest that the majority of young patients, especially women, report only slight or no exposure to these risk factors (8). Due to this apparent absence of significant habits in young people, factors such as immune deficiency, genetic factors and dietary factors have been considered as the main etiological agents (9), whilst the involvement of Herpes simplex virus and human papilloma virus have also been studied (10). However, HPV seems to be associated only with pharyngeal and oropharyngeal squamous cell carcinoma (2,9).

With respect to disease free and overall survival rates the data are even more inconsistent. In this regard, many authors refer to survival rates that are comparable with older patients (11,12). However, others have suggested that oral and oropharyngeal SCC show rapid disease progression and poorer prognosis in young people (13,14).

-Aim of the study

We conducted a retrospective analysis to examine a series of parameters such as sites of occurrence, tobacco and alcohol consumption, sex distribution, socio-economic status, T stage at diagnosis, degree of tumor differentiation, locoregional failure and overall survival at 5 years in Spanish patients aged < 45-years with oral and oropharyngeal SCC. In addition, we compared these results with a group of patients aged >45 years with oral and oropharyngeal SCC.

## Material and Methods

The medical records of 33 patients aged <45 years with oral and oropharyngeal SCC were retrospectively analyzed. These patients were diagnosed between 1998 and 2011 at the Granada University Hospital (Spain). During this time, 472 patients were diagnosed with oral and oropharyngeal SCC in this center. Thus, another 100 patients aged >45 years with oral and oropharyngeal SCC were randomly selected from the same database. We carried out a retrospective study to gather data on specific features such as sites of occurrence, risk factors, sex distribution, socio-economic status, T stage at diagnosis, nodal involvement, degree of tumor differentiation, locoregional failure and overall survival at 5 years. Finally, the outcomes of both groups were compared. SCC of the lip was excluded since it can originate from skin rather than mucosa. Furthermore, patients with an incomplete clinical history were also omitted.

We divided the sites into the tongue, floor of the mouth, buccal mucosa, alveolus, gingiva, and oropharynx. The World Health Organization classification was used to assess the histopathological type. The UICC TNM system for head and neck cancers was used to determine the features of the primary tumor, nodal involvement, and distant metastasis (15). The degree of differentiation of the tumor was defined according to the classification proposed by Bryne (16). Local recurrence was considered as the appearance of the same malignancy located in the vicinity of the primary tumor beds. Regional recurrence referred to cervical metastases diagnosed during follow-up (17). Socioeconomic status was established through the analysis of specific characteristics such as household income, education, and occupation (18). Patient follow-up was conducted with a clinical exploration every 3 months during the first year after surgery, every 6 months during the second and third years, and once per year thereafter.

The SPSS version of statistical software was used for data analysis. Descriptive statistics such as mean and standard deviations (SD) were used to calculate the average age of the patients. Frequency and percentages for sex distribution, sites, risk factors, socio-economic status, T stage at diagnosis, nodal involvement, degree of tumor differentiation, locoregional failure and overall survival at 5 years were calculated. The Chi-square was used to compare the difference between the two groups. The *P* value was set at 0.05. A Kaplan-Meier test was carried out for an overall 5-year survival analysis.

Further, we conducted a paired-matched analysis between the groups using a propensity score analysis. This test allows a pseudo-randomization of the groups based on definite variables. Specifically, patients were divided in five groups according with tumor site, clinical stage of disease and degree of tumor differentiation to reduce the heterogeneity of the participants. In addition, a logistic regression multivariate analysis was performed to consider the effect of confounding factors such as tumor site, clinical stage, degree of tumor differentiation and node.

## Results

-Socio-economic and demographic data

A total of 133 patients were identified. Of the group of patients aged >45 years, 67 were males and 33 were females. The male-female ratio was 2.03:1. Patient ages ranged between 46 and 97 years, with a mean of 64.32. In addition, we also observed that 67% of these patients came from a low social class with little education, 25 % were categorized as middle class, and 8 % upper class.

However, of the group of patients aged >45 years, 18 were males and 15 females. The male-female ratio was 1.2:1. Patient ages ranged between 19 and 45 years, with a mean of 33.71. In this group, 30. 3 % (n=10) came from a low social class with little education, 54.5 % (n=18) were categorized as middle class and 15. 1% (n=5) were upper class.

To summarize, in young people SCC is more frequent in patients from the middle-upper classes (*p*< 0,01). Moreover, the male-female ratio is more balanced than in the group aged >45 years.

-Risk Factors

87 % of patients >45 years of age had been smokers or drinkers throughout life. Among these, 69 % presented both risk factors, 13 % were only smokers and 5 % only drinkers. Thus, only 13% of patients aged >45 years had not reported exposure to alcohol and tobacco.

However, in the group of patients aged <45 years only 48.4% (n=16) were smokers throughout life. Among these, 25% (n=4) also reported high alcohol consumption. In contrast, 51.5 % (n=17) of young adults had no exposure to identifiable risk factors.

Our data therefore show that alcohol and tobacco have a poor correlation with SCC in young adults (*p*< 0,01).

-Site

In the group aged >45 years, the tongue was the area most commonly affected (35%, n=35). Other sites involved were the floor of the mouth (20%, n=20), the retromolar region (14%, n=14), buccal mucosa (9 n=9), oropharynx (8%, n=8), maxilla (7%, n=7), palate (5%, n=5), and gingiva (2%, n=2).

In the group aged <45 years, the tongue also represented the most affected area (54.5%, n=18). Other sites affected by SCC were the floor of the mouth (15,1%, n=5), buccal mucosa (12.1%, n=4), oropharynx (9.09%, n=3), alveolar ridge (3.03 %, n=1), maxilla (3.03 %, n=1), and gingiva (3.03 %, n=1).

In conclusion, the tongue was the most affected zone, with no significant differences between the two groups (*p*= 0,11).

-T stage at diagnosis

In accord with the UICC TNM system, T2 and T3 were the most frequent stages of presentation in patients aged >45 years with 37% (n=37) and 29% (n=29) respectively, whereas the rate of T1 and T4 was 27% (n=27) and 7% (n=7).

In contrast, T1 was the most frequent stage of presentation in patients aged <45 years (57.5%, n=19) followed by T2 (27.2%, n=9), T3 (3.03% n=2) and T4 (3.03% n=3) (*p*< 0.01).

-Nodal involvement (histological examination)

Nodal involvement was evident in 33% (n=33) of patients aged >45 years. However, in patients <45 years old, the neck lymph system was affected in 48.4% (n=16) of the cases. There was no significant association between nodal involvement and age (*p*=0,06).

-Degree of tumor differentiation

66% (n=66) of patients aged >45 years presented a moderately differentiated SCC, whilst well-differentiated and poorly-differentiated SCC were observed in 8% (n=8) and 26% (n=26) of cases respectively.

Moderately differentiated SCC was also the most common degree of differentiation in patients aged <45 years (51.5%, n=17). Well-differentiated SCC was evident in 39.3% of cases (n=13), whilst poorly differentiated SCC was observed in only 9.09% (n=3) of patients.

In short, moderately differentiated is the most common subtype of SCC in both groups. However, well-differentiated subtypes are more frequent in young patients (*p*< 0,01).

-Locoregional failure

The rate of locoregional failure was 34% (n=34) for patients aged >45 years, whilst young patients experienced locoregional failure in 45.4% of cases (n=15). In this regard, there was no significant association between locoregional failure and age (*p*=0.23).

-Overall Survival

An overall 5-year survival rate was estimated using the Kaplan–Meier survival analysis. With a mean follow up of 41.7 months (95 % CI: 37.5, 45.9), the 5-year overall survival rate for patients aged >45 years was 62% (n=62). However, the younger group had a mean follow up of 38.3 months (95 % CI: 29.9, 46.7) and a 5-year overall survival rate of 48.4% (n=16). There was no evidence of a significant association between overall survival and age (*p*=0,17).

**Figure 1:**
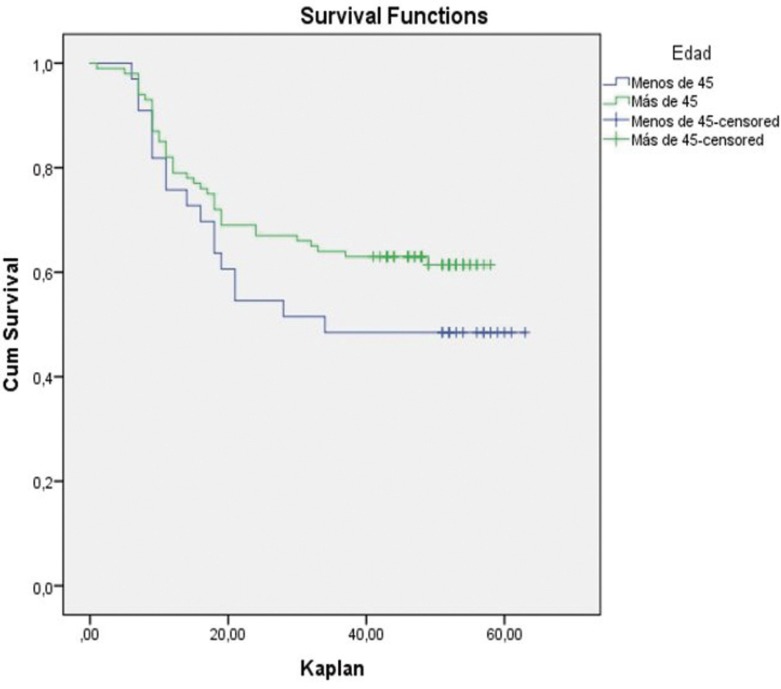
Survival analysis with Kaplan-Meier.

**Figure 2:**
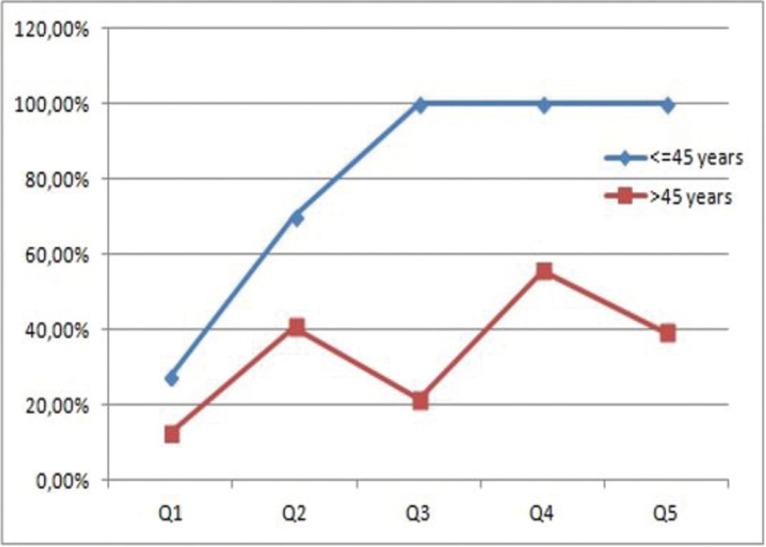
Mortality rate in each subgroup.

The paired-matched analysis showed worse prognosis for young people in all sub-groups ( Table 1, Table 2-Figs. 1,2). Unfortunately, due to the small sample of sub-groups, these results did not present statistical significance (*p*>0.05). In the same line, the logistic regression multivariate analysis evidenced an increased mortality rate in patients <45 years old (OR= 1.734; 0.789-3.832) and the adjustment for confounding factors (tumor site, clinical stage, degree of tumor differentiation and node) pointed out that node involvement (OR=3,183; IC 95% 1,430-7.083; *p* <0,05) and age <45 (OR= 3,9181, IC 95% 1.288-11.916; *p* <0,05) represent the major risk factors for patient mortality in our study ( Table 3).

**Table 1:**
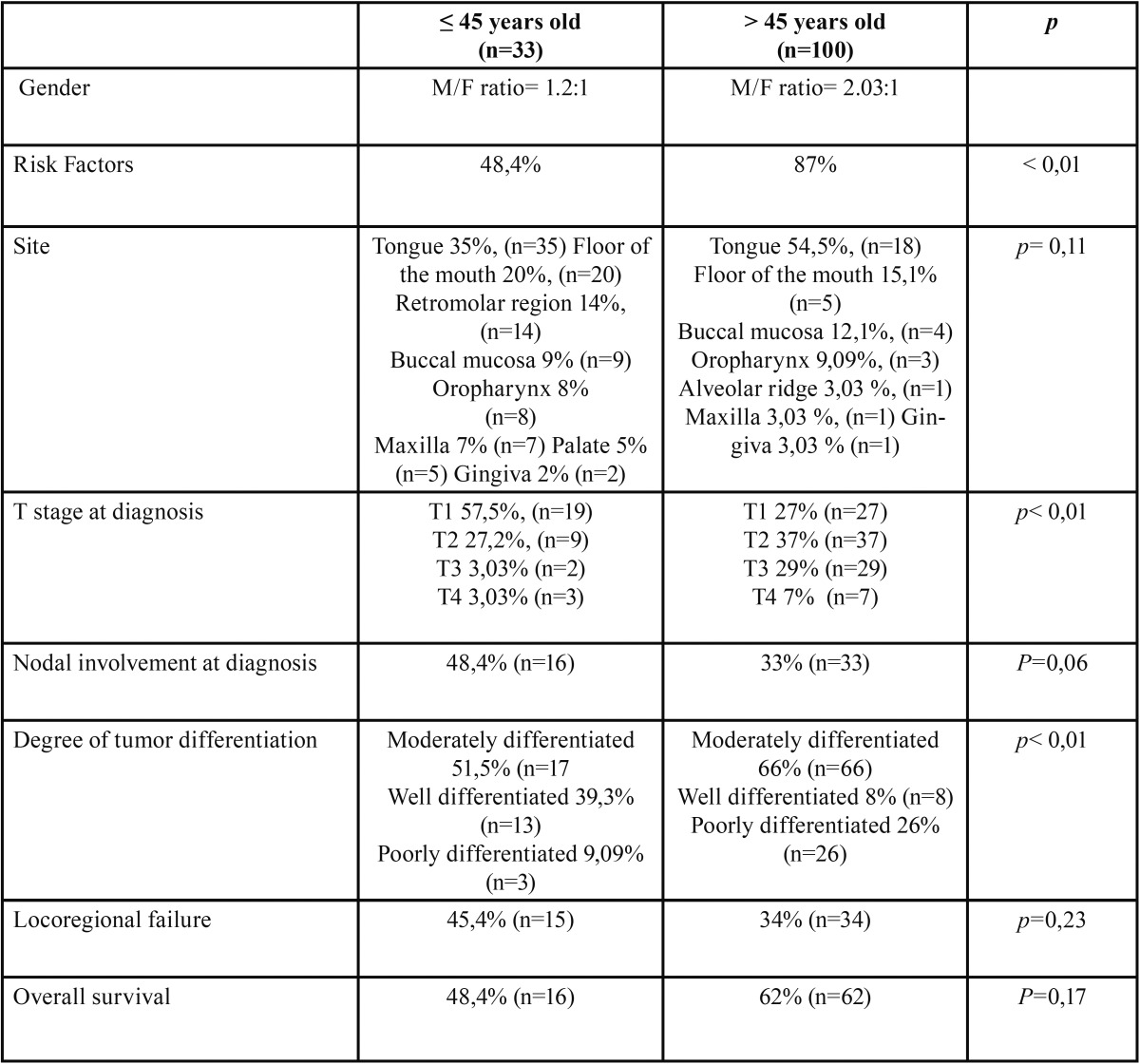
Results.

**Table 2:**
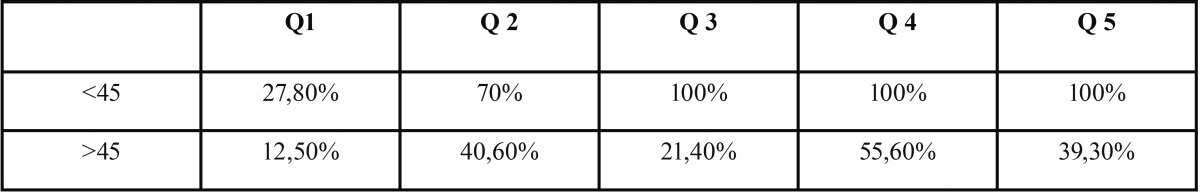
Paired-matched analysis. Young patients showed a higher mortality rate in in all subgroups.

**Table 3:**
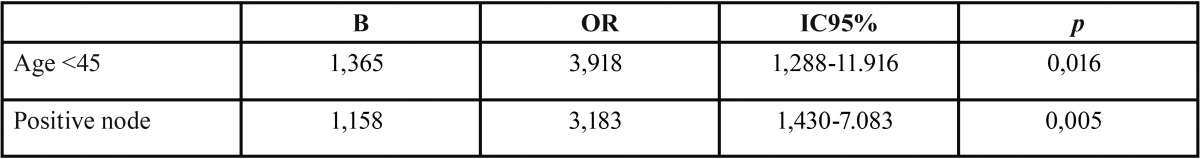
Logistic regression multivariate analysis. The prognosis of oral and oropharyngeal SCC is worse in patients aged < 45-years. The adjustment for confounding factors indicated that node involvement and age <45 years represent the major risk factors for patient mortality in our study.

## Discussion

Oral and oropharyngeal SCC are rare in young adults. In fact, patients below 45 years of age account for only 6% of oral and oropharyngeal SCC cases (14). However, many studies suggest that oral and oropharyngeal SCC is increasing in young adults worldwide (6,19-21). In particular, several reports have presented evidence for the rising incidence of tongue cancers in young people (2). The reasons for this increase are not known. Interestingly, numerous authors have noted only a weak correlation be-tween oral and oropharyngeal SCC in young people and typical risk factors such as tobacco and alcohol use (22). The relatively low exposure to these risks raises the possibility that other factors might be involved in the etiology of this disease (2). Our results also revealed that 51.5% of patients below the age of 45 reported no history of exposure to alcohol or tobacco. However, 87% of patients aged over 45 had been smokers or drinkers throughout life, a result that is compatible with the findings of other studies in the field.

The tongue appeared to be the most affected area in both groups, with the floor of the mouth representing the second-most affected region in the two groups. In contrast, SCC of alveolar ridge and maxilla are relatively rare in young people. These findings are also comparable with other outcomes reported in the literature (23,10).

Other relevant information is related to the degree of tumor differentiation. In this regard, our data show that moderately differentiated cancers were the most common subtypes in both groups. However, well-differentiated tumors were relatively more prevalent in the younger age group (39.3% vs. 8%), a trend that has also been observed by other authors (10,24). However, it is not known whether the degree of tumor differentiation could represent a marker of poor prognosis for young patients.

Importantly, the majority of the young patients (57,5%) presented T1 stage at the time of diagnosis, whereas patients aged >45 years showed a higher rate of T2 and T3. In accord with these data, the majority of other authors have reported a higher proportion of T1 and T2 tumors in young people (25). Thus, smaller cancers are more common in young patients. Nevertheless, our results revealed a higher rate of nodal involvement (48.4%) at presentation in the group of young adults ( Table 1) compared with a rate of only 33.3% in patients aged >45 years. In this respect, the literature is inconclusive, with the majority of studies being based on small samples. O’Regan *et al.* reported a rate of nodal involvement of 50% in patients aged <40 years (10), whilst Soon *et al.* reported a nodal involvement rate of higher than 50% (19). However, Sarkaria *et al.* described lower rates of nodal affectation in young patients. To be more specific, these authors studied the behavior of oral and oropharyngeal SCC in young people, analyzing 14 studies for a total of 132 patients aged <40 years. Interestingly, their results showed a high prevalence of early stage disease at presentation (64% stage I and II). In spite of this, the percentage of locoregional failure and overall survival was 57% and 47% (14). Hence, these results are comparable with our data. With regard to overall survival, our results revealed a poor prognosis for young people (*p*=0.17). However, it is important to note that the literature has yielded particularly inconsistent results in this field (5,7,24,26-28). Thus, the characteristics of SCC in young adults appear to be poorly understood. Unfortunately, the vast majority of studies are based on small samples and this has hindered progress in terms of advancing our knowledge of the phenomenon.

## Conclusions

SCC in young adults could have specific characteristics. In particular, the weak association with the common risk factors and the increasing prevalence of the disease in females suggest that other pathogenic mechanisms might be involved in this group of patients. Our results revealed that whilst the majority of young patients presented smaller tumors at diagnosis, the rate of nodal involvement at presentation was higher in this group. Similarly, young people showed poor outcomes in terms of locoregional failures and overall survival. Unfortunately, due to the restricted sample of almost all the studies, current knowledge regarding the characteristics of SCC in young people is limited, and the findings in the literature are often contradictory. Thus, considering that the incidence of OSCC is rapidly rising in young people, further research will be critical in order to improve the management of these cases.
